# Electrically tunable Berry curvature and strong light-matter coupling in liquid crystal microcavities with 2D perovskite

**DOI:** 10.1126/sciadv.abq7533

**Published:** 2022-10-05

**Authors:** Karolina Łempicka-Mirek, Mateusz Król, Helgi Sigurdsson, Adam Wincukiewicz, Przemysław Morawiak, Rafał Mazur, Marcin Muszyński, Wiktor Piecek, Przemysław Kula, Tomasz Stefaniuk, Maria Kamińska, Luisa De Marco, Pavlos G. Lagoudakis, Dario Ballarini, Daniele Sanvitto, Jacek Szczytko, Barbara Piętka

**Affiliations:** ^1^ Institute of Experimental Physics, Faculty of Physics, University of Warsaw, Pasteura 5, PL-02-093 Warsaw, Poland.; ^2^ Science Institute, University of Iceland, Dunhagi 3, IS-107 Reykjavik, Iceland.; ^3^ Department of Physics and Astronomy, University of Southampton, Southampton SO17 1BJ, UK.; ^4^ Institute of Applied Physics, Military University of Technology, Warsaw, Poland.; ^5^ Institute of Chemistry, Military University of Technology, Warsaw, Poland.; ^6^ Institute of Geophysics, Faculty of Physics, University of Warsaw, ul. Pasteura 5, PL-02-093 Warsaw, Poland.; ^7^ CNR-Nanotec, Institute of Nanotechnology, Via Monteroni, 73100 Lecce, Italy.; ^8^ Hybrid Photonics Laboratory, Skolkovo Institute of Science and Technology, Territory of Innovation Center Skolkovo, 6 Bolshoy Boulevard 30, Building 1, 121205 Moscow, Russia.

## Abstract

The field of spinoptronics is underpinned by good control over photonic spin-orbit coupling in devices that have strong optical nonlinearities. Such devices might hold the key to a new era of optoelectronics where momentum and polarization degrees of freedom of light are interwoven and interfaced with electronics. However, manipulating photons through electrical means is a daunting task given their charge neutrality. In this work, we present electrically tunable microcavity exciton-polariton resonances in a Rashba-Dresselhaus spin-orbit coupling field. We show that different spin-orbit coupling fields and the reduced cavity symmetry lead to tunable formation of the Berry curvature, the hallmark of quantum geometrical effects. For this, we have implemented an architecture of a photonic structure with a two-dimensional perovskite layer incorporated into a microcavity filled with nematic liquid crystal. Our work interfaces spinoptronic devices with electronics by combining electrical control over both the strong light-matter coupling conditions and artificial gauge fields.

## Teaser

The photonic Berry curvature in a strongly coupled liquid crystal cavity with 2D perovskite can be tuned by an electric field.

## INTRODUCTION

There has been surging interest from condensed matter and solid-state communities in generating artificial gauge fields across various platforms as a means to describe particle properties (*1*) such as cold atoms (*2*–*5*), photonic materials (*6*, *7*), acoustics (*8*), mechanical systems (*9*), and exciton-polariton cavities (*10*–*12*). Gauge fields play an important role in topological properties of matter (*13*, *14*) and can describe a fundamental band property known as the Berry curvature (*15*, *16*), quantifying the topological invariants of the system. The Berry curvature gives rise to an anomalous velocity term in a wave packet’s motion responsible for the Hall current (*17*) and the quantum Hall effect (*18*), with important implications in electronic transport (*19*).

In particular, artificial non-Abelian gauge potentials give rise to effective spin-orbit coupling (SOC) of particles, which has seen a lot of investigation recently in optics (*12*, *20*–*22*). SOC forms an important ingredient in the field of spintronics (*23*), and its optical analog spinoptronics based on cavity exciton-polaritons (*24*, *25*) seeks to exploit the mixture of internal (spin or polarization) and external (momentum) degrees of freedom for information processing. Exciton-polaritons (hereinafter just “polaritons”) arise in the strong light-matter coupling regime as mixed states of microcavity photons and excitons (*26*). They combine small effective photonic mass (∼10^−5^ of the electron mass) with strong nonlinear effects (*27*) and sensitivity to external fields provided by their excitonic matter component. Moreover, they present a unique opportunity over photonic systems to study nontrivial band geometry with the formation of the Berry curvature (*11*, *28*) and topological effects (*29*–*33*).

The most well-known photonic SOC in microcavities is the splitting of transverse electric and transverse magnetic (i.e., TE-TM splitting) cavity photon modes (*34*). This leads to a double winding effective magnetic field in the cavity plane that grows quadratically in the photon in-plane momentum (*35*). Recently, the photonic analog of the electronic Dresselhaus (*36*) and Rashba-Dresselhaus (RD) (*37*, *38*) SOCs have been realized in microcavities. For the case of cavities that host both RD SOC and TE-TM splitting, it has been shown theoretically that reducing the cavity symmetry could form local concentrations of the Berry curvature (*39*) without the need to break time-reversal symmetry through external magnetic fields acting on the excitonic component of polaritons (*11*, *40*, *41*) or nonzero optical activity for the photons (*28*, *42*).

## RESULTS

In this work, we present a method to electrically tune a photonic Berry curvature in the strong light-matter coupling regime. Our optical system is composed of a liquid crystal (LC) cavity where both RD SOC and TE-TM splitting effects of the cavity photons are inherited in the emerging exciton-polariton modes because of an additional cavity-embedded perovskite layer. The strongly bound perovskite excitons allow the observation of strong coupling at room temperature with a high quantum yield and nonlinear effects up to four orders of magnitude higher than in other photonic systems (*12*, *43*) and thus present a good material to be interfaced with LCs. Because of the high birefringence and electric permittivity anisotropy of LCs, making them sensitive to external electric fields, we achieve unprecedented electric control over an emerging polaritonic Berry curvature through the specially synthesized photonic SOC and reduced cavity symmetry.

**Fig. 1.**
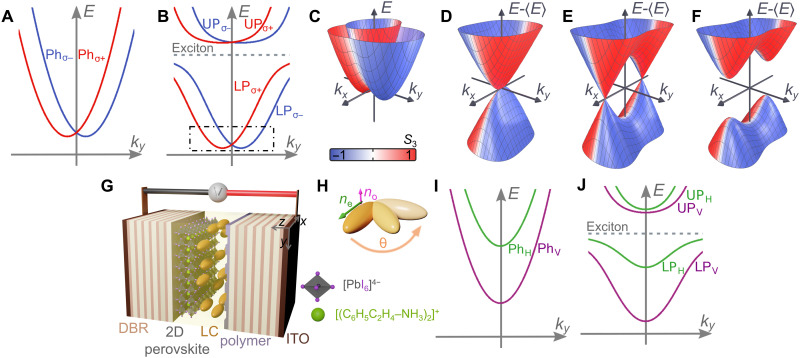
LC microcavity with 2D perovskite. Schematic dispersion relation of (**A**) bare cavity photon modes in the RD SOC regime and (**B**) in the strong light-matter coupling regime. (**C**) Dispersion relation for the bottom of the lower polariton branch [the region marked by a dot-dashed rectangle in (B)]. (**D**) Same as (C) with the dispersion subtracted by its mean value ⟨*E*⟩ to more clearly show the intersection points. Energy of the modes for (**E**) positive horizontal-vertical (H-V) splitting and (**F**) with broken inversion symmetry. (**G**) Schematic representation of the LC microcavity with the two-dimensional (2D) hybrid organic-inorganic perovskite layer and LC molecules oriented parallel to the *x*-*y* plane at zero voltage. (**H**) Rotation of an LC molecule under applied voltage with refractive indices (*n*_o_, *n*_e_) and angle θ in the *z*-*x* plane. We also show the bare photon (**I**) and polariton (**J**) dispersions in the linear H-V polarization basis in the absence of voltage to highlight their splitting. ITO, indium tin oxide.

A cross section of the photonic RD dispersion in the cavity (*k_x_, k_y_*) plane is schematically shown in Fig. 1A, depicting two opposite circularly polarized valleys in analogy with spin ^1^/_2_ systems. When the photons become strongly coupled with the perovskite excitons, a characteristic anticrossing behavior occurs, as shown in Fig. 1B, where the low-energy polariton modes (within the dot-dashed rectangular box) adopt approximately the same dispersion as that of the photons (Fig. 1A). A surface plot of the polariton dispersion at low momenta is shown in Fig. 1C. A single degeneracy point at normal incidence (**k** = 0) is highlighted in the RD polariton dispersion (Fig. 1D), where we have subtracted the dispersion from its mean ⟨*E*⟩. When TE-TM splitting and an additional uniform in-plane effective magnetic field **B***_x_* = ∆_HV_$\hat{x}$ are present, the RD degeneracy point morphs into two Dirac cones with degeneracy points known as diabolical points (Fig. 1E) (*11*, *33*, *42*, *44*, *45*). The rapidly whirling RD SOC field around these points leads to a topologically trivial gap opening (Fig. 1F) when an additional perpendicular **B***_y_* = ∆_AD_$\hat{y}$ effective magnetic field is introduced to break the inversion symmetry of the system with subsequent formation of a Berry curvature dipole (as shown later in the text).

### Strong light-matter coupling

The microcavity consists of two distributed Bragg reflectors (DBRs) facing each other with an embedded 60-nm-thick polycrystalline two-dimensional (2D) phenylethylammonium iodide (PEAI) perovskite (C_6_H_5_C_2_H_4_NH_3_)_2_PbI_4_ (hereinafter PEPI), shown schematically in Fig. 1G. The perovskite polycrystalline thin film was prepared on one of the DBR inner sides using a spin-coating method (*46*). The cavity structure was designed to enhance the photonic field at the position of the perovskite layer. The cavity was also filled with a highly birefringent nematic LC that acts as a uniaxial medium with ordinary *n*_o_ and extraordinary *n*_e_ refractive indices [∆*n* = *n*_e_ − n_o_ = 0.4; (*47*)]. The voltage applied to transparent electrodes [made of indium tin oxide (ITO)] rotates the molecular director, hence the direction of the optical axis of the LC medium, by an angle θ in the *x*-*z* plane (see Fig. 1H). This enables direct control over the cavity effective refractive indices. Throughout the paper, we will refer to the Stokes parameters of the emitted cavity light (analogous to the polariton pseudospin) as *S*_1_ = (*I*_H_ − *I*_V_)/(*I*_H_ + *I*_V_), *S*_2_ = (*I*_D_ − *I*_AD_)/(*I*_D_ + *I*_AD_), and *S*_3_ = (*I*_σ*+*_ − *I*_σ−_)/(*I*_σ*+*_ + *I*_σ−_), corresponding to intensities of horizontal (*I*_H_), vertical (*I*_V_), diagonal (*I*_D_), antidiagonal (*I*_AD_), right-hand circular (*I*_σ+_), and left-hand circular (*I*_σ−_) polarized light.

The alignment of the LC molecules inside the cavity at zero voltage is determined by an ordering polymer layer rubbed along the *x* (H) direction, as shown schematically in Fig. 1G. Consequently, the bare photon dispersion in the linear horizontal-vertical (H-V) polarization basis shown in Fig. 1I displays strongly split bands described by the Hamiltonian

$${\hat{H}}_{\phi }(k,\theta )=\frac{\hbar^{2}}{2}(\frac{k_{x}^{2}}{m_{x}}+\frac{k_{y}^{2}}{m_{y}})+\hat{S}(k)+\frac{\Delta_{\text{HV}}}{2}{\hat{\sigma }}_{z}$$ (1)

where **k** = (*k_x_, k_y_*)^T^ is the in-plane momentum. Here, $\hat{S}$ describes an effective photonic SOC coming from both cavity TE-TM splitting (*34*) and the LC anisotropy (*37*)

$$\hat{S}=2\delta_{\mathrm{xy}}k_{x}k_{y}{\hat{\sigma }}_{x}+(\delta_{x}k_{x}^{2}-\delta_{y}k_{y}^{2}){\hat{\sigma }}_{z}$$ (2)

Physically, this operator introduces direction-dependent modification to the effective masses of linearly polarized modes. When δ*_xy_* = δ*_y_* = δ*_x_*, it realizes the conventional TE-TM splitting (*48*). Last, ∆_HV_ describes uniform splitting between H- and V-polarized modes (sometimes referred as *X*-*Y* splitting), which can be directly controlled in the experiment through the voltage applied to the ITO electrodes on the cavity, where the electric field drives the molecular director, hence the optical axis, and changes the effective refractive indices for the H-polarized mode. We note that all coefficients in Eqs. 1 and 2 depend on θ but not as strongly as ∆_HV_ (*37*). We do not consider non-Hermitian effects that are weak in our system and only relevant to the physics of exceptional points (*33*, *49*, *50*), which is beyond the scope of our study.

When an exciton resonance, with energy *E*_χ_, from the perovskite layer is introduced to the cavity (dashed horizontal line in Fig. 1J), the linearly polarized photonic modes coherently couple to excitons at a rate defined by the Rabi energies Ω_H,V_. The strongly coupled system can be described by a coupled oscillator model represented by a Hamiltonian

$${\hat{H}}_{\text{SC}}=(\begin{array}{cc} E_{\chi } & 0\\ 0 & 0 \end{array})⊗I_{2}+(\begin{array}{cc} 0 & 0\\ 0 & 1 \end{array})⊗{\hat{H}}_{\phi }+\frac{{\hat{\sigma }}_{x}}{2}⊗(\begin{array}{cc} \Omega_{H} & 0\\ 0 & \Omega_{V} \end{array})$$ (3)

The four polariton eigenmodes are shown in Fig. 1J, exhibiting characteristic anticrossing behavior, and labeled as upper (UP_H,V_) and lower polaritons (LP_H,V_).

**Fig. 2.**
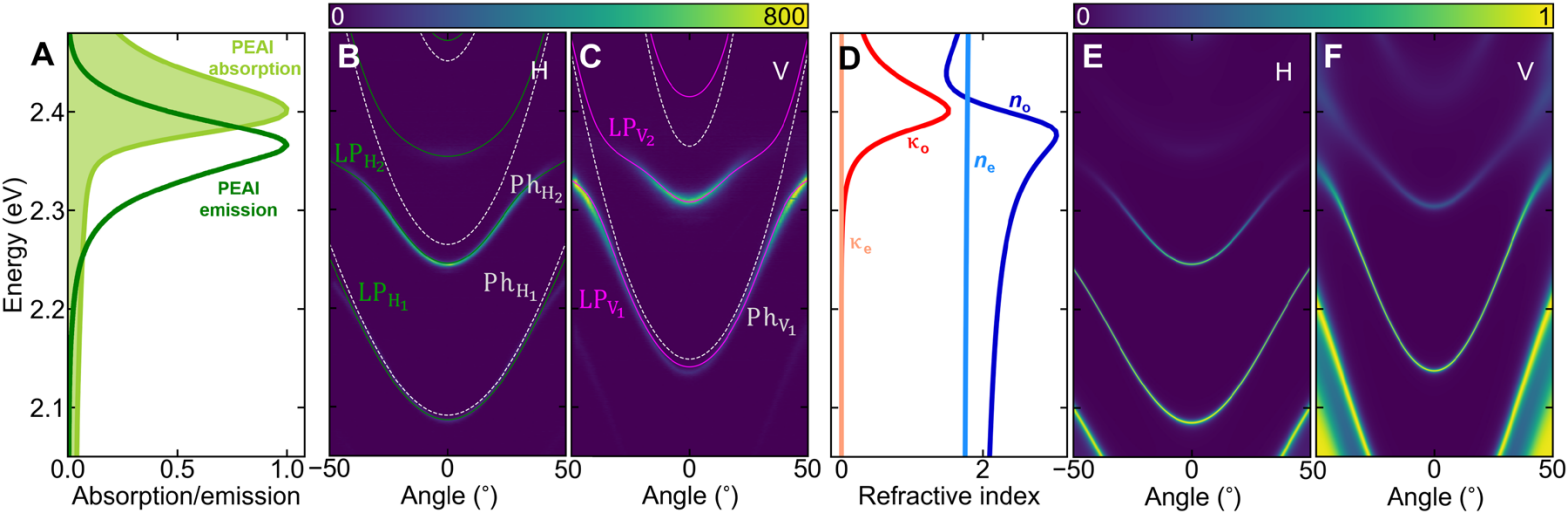
Strong light-matter coupling regime in LC microcavity with 2D perovskite. (**A**) Normalized absorption (light green line) and emission (dark green line) spectra of a polycrystalline 2D perovskite based on PEAI, phenylethylammonium iodide. (**B** and **C**) Angle-resolved photoluminescence spectra showing the strong-coupling dispersion in H and V polarization at zero voltage (θ=0). White dashed curves indicate calculated bare photon dispersion, and green and magenta curves denote the calculated polariton dispersion (see Materials and Methods). (**D**) Real *n*_o,e_ and imaginary κ_o,e_ parts of the ordinary and extraordinary refractive indices for a thin polycrystaline 2D perovskite layer. (**E** and **F**) Berreman simulations corresponding to (B) and (C) (see Materials and Methods).

The strong coupling regime in our structure is illustrated in Fig. 2. The strong emission (dark green line) and absorption (light green line) spectra of a thin PEPI layer without the cavity are shown in Fig. 2A. The maximum of the spectra overlap is at 2.38 eV, which corresponds to the most effective emission and reabsorption processes. The emission spectra from our cavity at room temperature at zero voltage (i.e., θ = 0) are presented in Fig. 2 (B and C), showing strong emission from H-V polariton modes. The characteristic anticrossing behavior between the excitonic resonance and the cavity photon modes is visible in the emission spectra at high emission angles.

Our measurements are compared with the solutions of Eq. 3 plotted with solid lines in Fig. 2 (B and C) (see Materials and Methods). The white dashed lines indicate the fitted bare photonic branches. Extracted Rabi energies for H- and V-polarized modes are Ω_H_ = 94.4 meV and Ω_V_ = 108.7 meV, respectively. This difference between the Rabi energies is due to different optical paths for the two linear polarizations (difference in the LC refractive indices *n*_o_ and *n*_e_).

We also performed numerical simulations on the optical properties of our cavity using the Berreman matrix method. For this purpose, the real and imaginary parts of the ordinary and extraordinary refractive indices *n*_o,e_ and κ_o,e_, respectively, were obtained from ellipsometric measurements and are presented in Fig. 2D. We observe a slight birefringence of the polycrystalline perovskite in the *z*-axis direction (perpendicular to the cavity plane). The simulated angle-resolved transmission spectra are shown in Fig. 2 (E and F). The theoretical result is fully consistent with the experiment. The higher-energy modes (above 2.39 eV) are not visible in the experimental spectra due to the strong absorption of the perovskite in this spectral range.

### RD polaritons

By rotating the molecular director of the LC with applied voltage, photonic modes of different polarization and parities become mixed and form an RD SOC dispersion relation (*37*). In this case, the photonic modes are dominantly circularly polarized, forming a dispersion depicting two shifted valleys of the opposite circular polarization (as shown in Fig. 1A). In this regime, the photonic Hamiltonian can be written as

$${\hat{H}}_{\phi }^{\text{RD}}(k,\theta )={\hat{H}}_{\phi }(k,\theta )-2\alpha k_{y}{\hat{\sigma }}_{y}$$ (4)

where α is the strength of the RD SOC. Note that, in this low-energy regime, polariton and photon lasing was recently demonstrated (*51*, *52*).

**Fig. 3.**
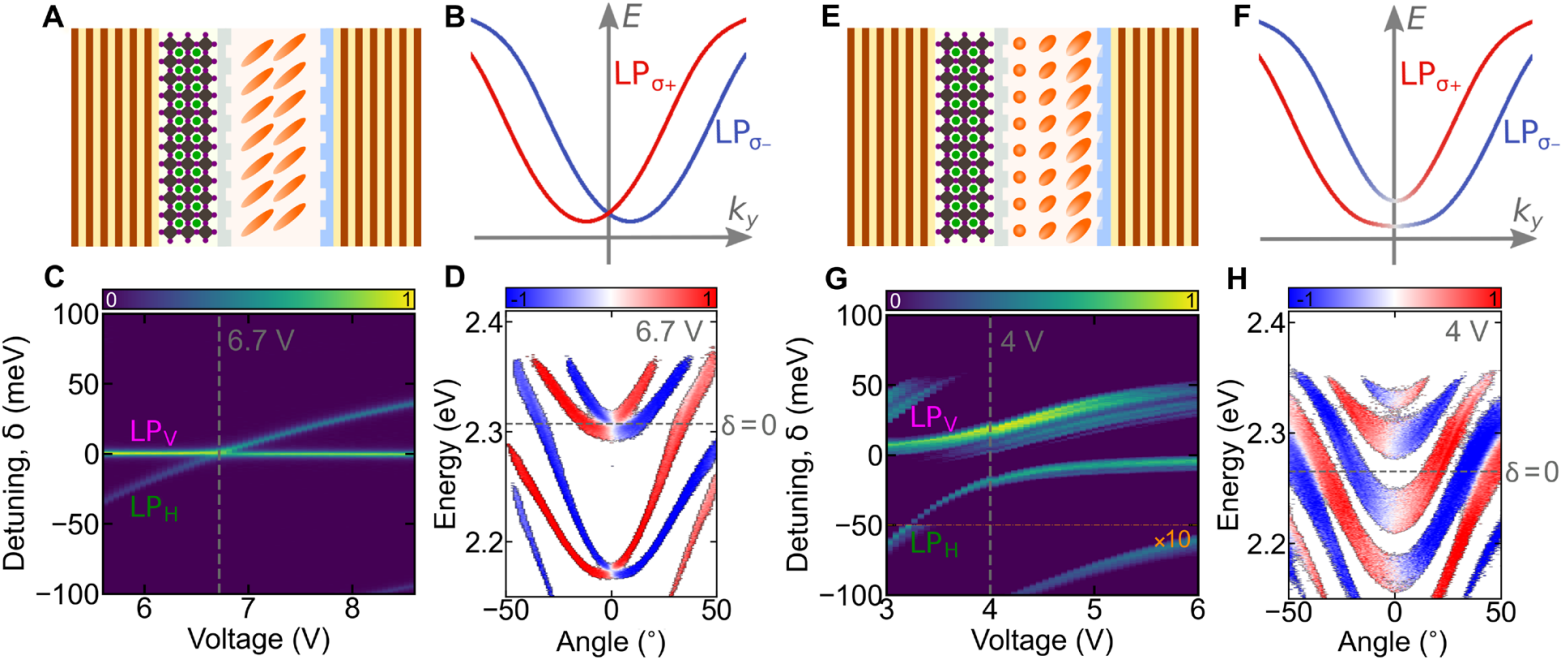
Gap opening in the RD regime. (**A** and **E**) Scheme of a 2D polycrystalline perovskite microcavity with an LC having two parallel ordering polymer layers at both sides of the cavity (A) and two ordering polymer layers at both sides of the cavity with 45° tilted rubbing (E) (see Materials and Methods). (**B** and **F**) Dispersion relation of first polariton branch in the RD SOC regime (B), with the opening of the energy gap (F). (**C** and **G**) Measured energy-resolved PL at **k**=0 versus applied voltage for diagonal polarization, without (C) and with (G) gap opening. (**D** and **H**) Measured angle-resolved PL spectra in the R-D regime for *S*_3_ polarization: without (D) and with (H) gap opening.

The polariton RD SOC regime is illustrated in Fig. 3 for two different cavity types. In Fig. 3 (A to D), we show the effects of RD SOC photonic modes coupled to the excitonic resonance. Here, the LC molecules are initially (at 0 V) oriented by two parallel rubbing polymer layers as shown in Fig. 3A.

The degeneracy point in the RD dispersion (see Fig. 3B; **k** = 0) is sensitive to H-V splitting, which can be directly controlled through the applied voltage [i.e., ∆_HV_ (θ)]. We measured the cavity PL as a function of applied voltage at **k** = 0, shown in Fig. 3C, and observed the crossing of two polariton modes at around 6.7 V. This crossing point corresponds to the degeneracy point in the RD polariton dispersion, which is evidenced through the measured *S*_3_ component shown in Fig. 3D.

### Berry curvature dipole

Synthesizing polaritonic Berry curvature in optical cavities is a challenging task because it requires breaking time-reversal symmetry through external magnetic fields acting on the excitonic component (*11*, *28*, *40*, *41*) or nonzero optical activity for the photons (*42*). Such chiral terms open a gap at the diabolical points in the polariton dispersion, corresponding to a Dirac cone intersection (*12*, *44*, *53*, *54*), where the TE-TM SOC field from $\hat{S}$ and the uniform field ∆_HV_ cancel each other out. With α = 0, the polariton modes are linearly polarized with a rapidly changing in-plane SOC field winding around the Dirac cones [i.e., the polariton pseudospin winds around the *S*_1_-*S*_2_ plane when going around the diabolical point (*11*, *12*)]. It is worth noting that when non-Hermitian terms are present, these diabolical points become exceptional points that expand to degeneracy lines (Fermi arcs) when polarization-dependent losses are present (*33*, *50*, *55*, *56*).

When RD SOC is present, it introduces an additional rapidly varying field around the Dirac cones manifested in the strong *S*_3_ component in the polariton dispersion. Practically, the diabolical points can only appear along the *k_y_* = 0 direction where the RD field vanishes (see Fig. 1E). Therefore, the RD field will only affect the SOC field around these points, but it cannot open the gap. However, because of the combined TE-TM and RD SOC fields, the polariton pseudospin no longer winds just in the *S*_1_-*S*_2_ plane when going around the diabolical points but also around the *S*_1_-*S*_3_ plane. This means that an effective magnetic field in the *y* direction corresponding to a finite *S*_2_ pseudospin component will break the inversion symmetry at the diabolical points and open the gap. Physically, such an effective magnetic field corresponds to splitting between the D and AD modes written as

$${\hat{H}}_{\phi }^{\text{RD}}(k,\theta )={\hat{H}}_{\phi }(k,\theta )-2\alpha k_{y}{\hat{\sigma }}_{y}+\frac{\Delta_{\text{AD}}}{2}{\hat{\sigma }}_{x}$$ (5)

Setting *k_y_* = 0, it can be shown that the presence of the ∆_AD_ breaks the parity inversion symmetry ${\hat{\sigma }}_{z}{\hat{H}}_{\phi }^{\text{RD}}(-k_{x},0){\hat{\sigma }}_{z}\neq {\hat{H}}_{\phi }^{\text{RD}}(k_{x},0),$ where ${\hat{\sigma }}_{z}$, in HV basis, flips the photon circular polarization. This leads to the opening of the gap with subsequent formation of local dispersion relation describing massive Dirac particles (see Fig. 1F).

**Fig. 4.**
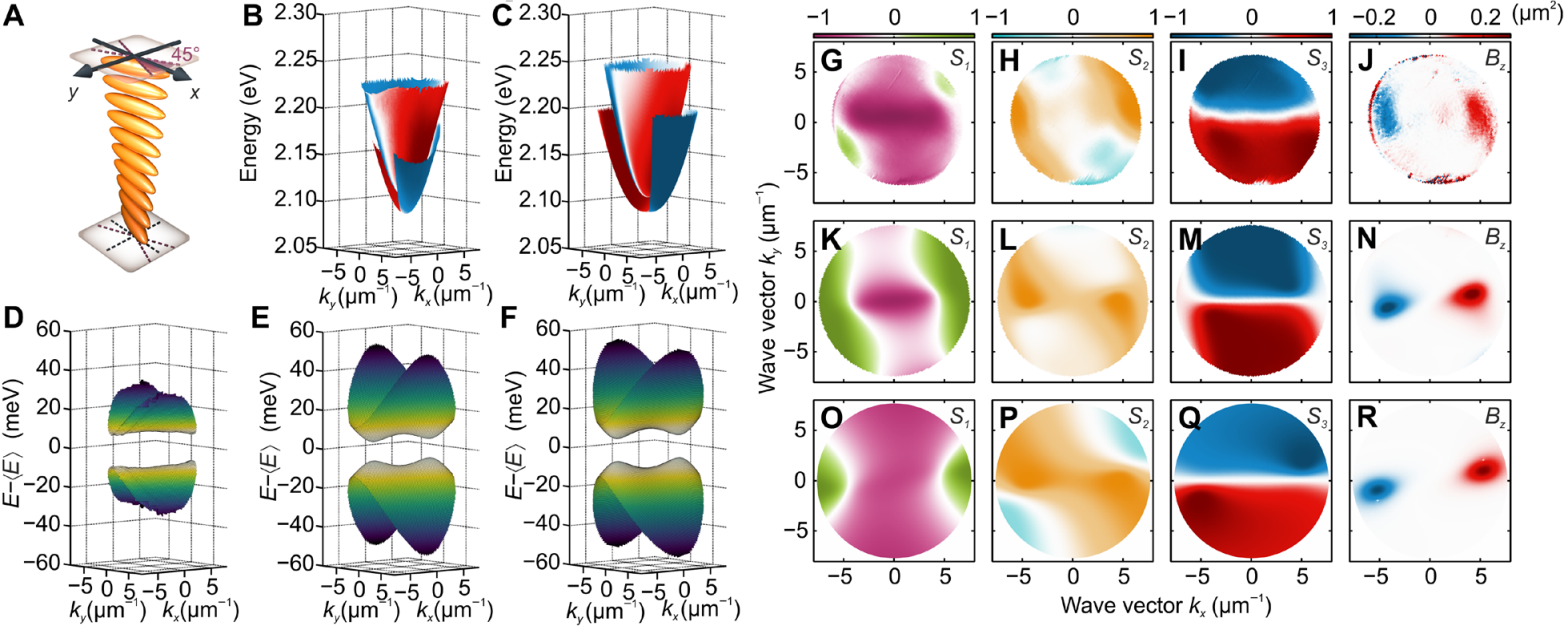
Nonzero Berry curvature in the LC cavity with 2D perovskite. (**A**) Scheme of orientation of LC molecules inside the cavity in twisted nematic configuration. Dispersion of polaritonic modes obtained by (**B**) experiment and (**C**) Berreman matrix simulations. The pseudocolor scale denotes the *S*_3_ component. (**D**) Measured polariton bands subtracted from their mean energy with corresponding (**E**) Berreman matrix simulations and (**F**) solutions of the analytical model. (**G** to **J**) Experimental polarization and resulting Berry curvature dipole of the lower-energy band compared with (**K** to **N**) results of the Berreman simulations and (**O** to **R**) the analytical two-level model.

For this purpose, we have prepared a second sample with rubbing at the perovskite layer oriented at an angle of 45° with respect to the rubbing at the DBR surface (Fig. 3E). This results in a twisted nematic LC structure (as illustrated in Fig. 4A), which introduces a deterministic D-AD splitting. The splitting is confirmed in Fig. 3G, where the PL spectrum at **k** = 0 as a function of voltage shows a clear anticrossing. Figure 3H shows the circular polarization degree of the corresponding angle-resolved dispersion, and Fig. 3F shows the calculated dispersion.

The concave energy relation indicating the location of the massive Dirac cones with a clear splitting is illustrated in Fig. 4 (B to F). The Stokes (pseudospin) parameters in Fig. 4 (G to I) show that, at the minimum of energy gap, the *S*_1_ and *S*_3_ polarizations of the bands whirl around two diagonally polarized points located along *k_x_*. This shows that the splitting between D- and AD-polarized modes in the twisted nematic configuration has opened a gap at these points. These points are accompanied by the strong concentration of the polaritonic Berry curvature as measured and calculated in Fig. 4 (J, N, and R). As the time-reversal symmetry that is conserved in the system integral over the Berry curvature within a single band is equal to zero, for the second band, the Berry curvature is expected to be of the opposite sign. We note that, at **k** = 0 in Fig. 4G, the mode is predominantly V polarized due to the applied voltage causing some additional splitting ∆_HV_. All of our results are in qualitative agreement with both our analytical model of Eq. 5 and Berreman simulations. Results of the analytical model in Fig. 4F were fitted to the dispersion relation obtained from the Berreman matrix model (see Materials and Methods).

**Fig. 5.**
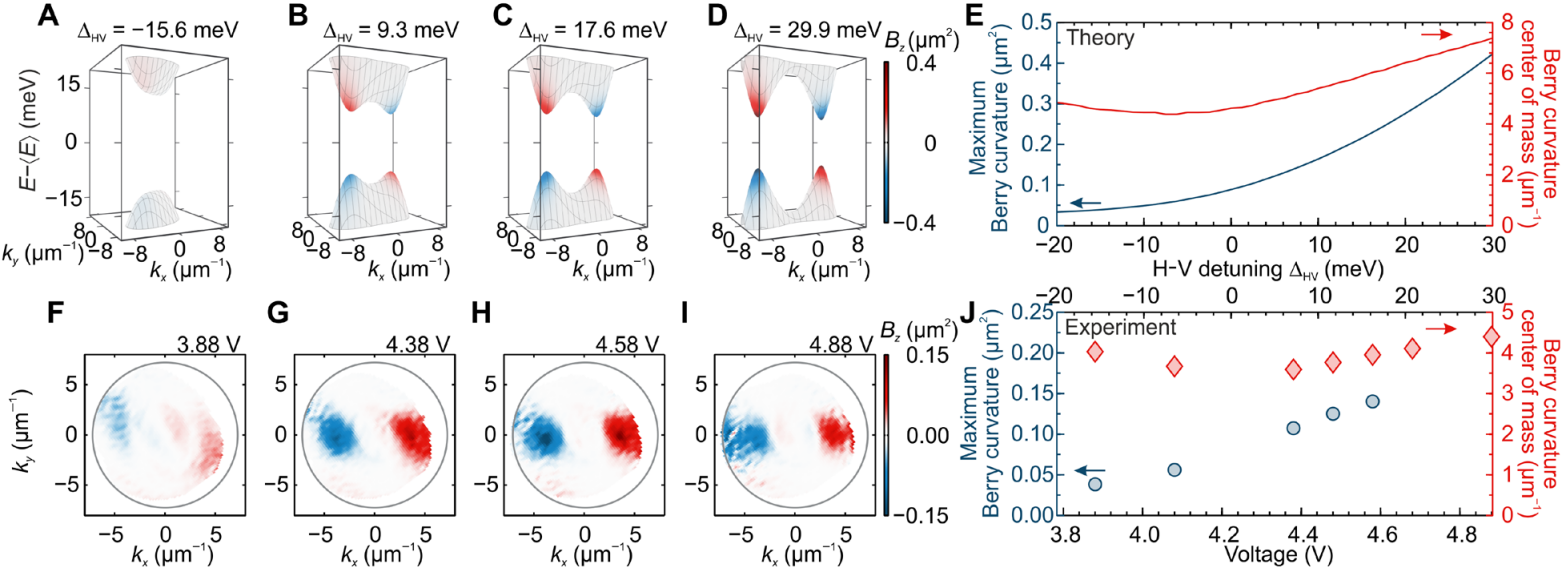
Electrical tuning of polariton Berry curvature. (**A** to **D**) Calculated dispersion of the cavity modes (energies subtracted by their mean) with *B_z_* marked by a pseudocolor scale from the analytical model for increasing H-V detuning ∆_HV_. (**E**) Resulting maximum value and center of mass position of the *B_z_* in reciprocal space for varying H-V detuning. (**F** to **I**) Experimental *B_z_* distribution for lower-energy mode with increasing voltage. (**J**) Experimentally measured maximum value of *B_z_* and *B_z_* center of mass position depending on applied voltage.

Last, the observed polariton Berry curvature can be electrically tuned through the parameter ∆_HV_, which is directly controlled by the amplitude of the external voltage applied to the cavity. Figure 5 (A to E) presents calculations based on Eq. 4 for varying detuning between the H and V modes. The calculated energies of the two bands are subtracted from their mean value, with the Berry curvature *B_z_* marked by a pseudocolor scale. The Berry curvature increases with H-V detuning as the dispersion relation changes to two gapped Dirac cones exhibiting a pronounced Berry curvature. Dependence of the maximum value of Berry curvature and the *B_z_* center of mass position in reciprocal space is summarized in Fig. 5E, which shows that the momentum position of the Berry curvature maximum can be controlled by ∆_HV_ detuning and can be effectively switched off for negative detunings.

Those theoretical predictions can be confirmed experimentally for varying voltage applied to the cavity that directly controls ∆_HV_ detuning. Figure 5 (F to I) presents Berry curvature extracted from polarization-resolved transmission measurement at different voltages applied to the ITO electrodes of the cavity. At 3.88 V (Fig. 5F), corresponding to negative detuning, the observed value of the Berry curvatures is low but rapidly increases for higher voltages when ∆_HV_ detuning becomes positive (Fig. 5G). Further increase of the applied voltage shifts the position of the *B_z_* maxima to higher momenta in reciprocal space up to the limits of the numerical aperture in the experiment (Fig. 5, H and I). The experimental position and value of the polaritonic Berry curvature summarized in Fig. 5J are in good agreement with theoretical predictions of Fig. 5E and demonstrate the electric tunability of the band geometry in our system. The polaritonic Berry curvature in our system is tunable in a continuous manner with the amplitude of external voltage, rather than the magnetic field or temperature (*28*).

## DISCUSSION

In conclusion, we have presented a novel architecture for a photonic cavity heterostructure with a 2D perovskite incorporated in a highly birefringent medium. The medium is a nematic LC that can be arranged in various ways into the cavity through proper preparation of embedded ordering layers. We first achieve the strong light-matter coupling regime between the perovskite excitons and cavity photons in the electrically tunable microcavity at room temperature with Rabi splitting in the order of ~100 meV. We demonstrate how the extreme birefringence coming from the LC results in exciton-polariton modes following an RD SOC dispersion relation, which opens exciting perspectives on designing nonlinear valley-optronic devices.

Second, the reduced symmetry of the cavity structure is found to open a gap at polaritonic diabolical points corresponding to Dirac cones in the cavity dispersion relation. This allows us to engineer local concentrations of Berry curvature that can be controlled through applied external voltage, which has only been possible using external magnetic fields or temperature variations (*28*). The importance of the Berry curvature dipole to the quantum nonlinear Hall effect (*57*) and rectification in polar semiconductors (*58*) was recently stressed in a study demonstrating its electric tunability in a monolayer of WTe_2_ (*59*). The proposed scheme to obtain non-zero Berry curvature can also be used in purely photonic systems such as LC cavities incorporating organic dyes (*52*), but here, we additionally operate in a strong coupling regime. Our work therefore takes steps toward interfacing polariton spinoptronic technologies with electronics at room temperature where nontrivial geometrical properties of the polariton dispersion can be easily tuned through applied voltage. We note that both electrically pumped polariton lasers (*60*) and electro-optic–modulated polariton topological lasers (*61*) are already possible at cryogenic temperatures. Moreover, with already exciting work aimed at RD polariton condensation (*51*), we expect that our platform can serve as a testbed for driven dissipative quantum matter in unconventional artificial gauge fields.

## MATERIALS AND METHODS

### Preparation of LC microcavity with 2D perovskite

The studied microcavity structure consists of two DBRs made of six SiO_2_/TiO_2_ (SiO_2_ top layer) pairs with a maximum reflectance at 520 nm. DBRs were grown on glass substrates with ITO electrodes. The surface of the dielectric mirror was cleaned with isopropanol and acetone to remove organic residues from the surface. It was then activated in an oxygen plasma. Because of the activation of the DBR surface, the 2D perovskite crystallization precursor solution adheres better to the substrate. The precursor solution was prepared in a glove box under an argon atmosphere as follows: PEAI was mixed with lead iodide (PbI_2_) in a stoichiometric 2:1 molar ratio and dissolved in *N,N*-dimethylformamide (mass percent: 10%). The solution was stirred for 4 hours at 50°C. All reagents were purchased from Sigma-Aldrich. Forty microliters of solution containing PEAI and PbI_2_ was spin-coated in air on a dielectric mirror with a rotation speed of 2000 rpm for 30 s. Approximately 60-nm-thick 2D-layered polycrystalline lead iodide perovskite [(C_6_H_5_(CH_2_)_2_NH_3_)_2_PbI_4_] was obtained upon solvent evaporation on the hot plate for 1 min at 100°C.

There were two types of microcavities as shown in (Fig. 3). For these samples, the preparation of the dielectric mirror coated with 2D polycrystalline perovskite was the same, as described above. In the case of the first sample (Fig. 3A), the bottom dielectric mirror with perovskite was spin-coated with a thin, protective layer (50 nm thick) of poly(methyl methacrylate) (PMMA) with a 3% methoxybenzene solution. The upper dielectric mirror was covered with a structured polymer-orienting layer of SE-130. The space between the DBRs is filled with a highly birefringent LC of ∆*n* = 0.4. Both mirrors were rubbed to obtain homogeneous LC orientation within the cavity. The total thickness of this LC cavity was approximately 3 μm. The second sample type was similar in design to the first one, but the PMMA layer on the dielectric mirror was arranged at an angle of 45° (Figs. 3, E, G, and H, and 4) or 30° (Fig. 5) to the rubbed layer of SE-130 polymer. The thickness of those cavities was approximately 2.5 μm. The different thickness of the presented three perovskite LC cavities gives a different range of LC voltage tuning to the RD regime (Fig. 3, C and G, and figs. S7 and S8).

### Optical measurements

Figure S10 shows the scheme of the experimental setup used for the optical measurements in both configuration: reflectance and transmission. The measurements were performed at room temperature using a continuous-wave laser at 405 nm for photoluminescence (PL) excitation and a white light lamp for the reflectance and transmission spectra. PL measurements were realized in the reflection configuration.

### Absorbance measurements

Absorption measurements of the PEPI perovskite on the glass substrate were performed using a Cary 5000 UV-Vis-NIR spectrometer. The absorption spectrum is presented in Fig. 2A.

### Ellipsometry measurements

The refractive index of polycrystalline PEPI perovskite (presented in Fig. 2D) was estimated on the basis of ellipsometric data from an RC2 ellipsometer (J.A. Woollam Company) in the 450 to 1690 nm spectral range. To increase the sensitivity of the measurement and retrieve anisotropy more accurately, two different types of samples were prepared. In the first set of samples, PEPI was spin-coated on the silicon wafer with a 618-nm-thick layer of thermal oxide to use the interference enhancement effect. Then, all 16 Mueller matrix (MM) elements in reflection were acquired for angles of incidence from 55° to 70° by 5°. In the second case, the PEPI layer was spin-coated on a 1-mm-thick transparent fused silica substrate. For this set of samples, transmission ellipsometry and transmission intensity data for illumination angles ranging from 0° to 40° by 5° were acquired additionally. Such a combination helps to reduce the correlation between the model parameters, leading to a unique solution. Almost negligible values of off-diagonal terms and off-diagonal blocks in the MM data collected in the reflection mode reveal no cross-polarization between *p*- and *s*-states and indicate that the sample under investigation is either isotropic or uniaxial with a *c*-plane anisotropy. As the sample rotation does not influence the MM elements, we assume that the *c* axis is perpendicular to the sample surface. All further data analysis was performed using the CompleteEASE software. The datasets were combined in a multisample model with the same optical constants for each PEPI layer. We used the general oscillator approach to retrieve the dielectric permittivity function constrained with Kramers-Kronig consistency. Typically, the presence of anisotropy alters the shape of features in the ellipsometric data. In the studied case of PEPI layers, incorporation of anisotropy into the model leads to 75% improvement of the mean square error of the dielectric permittivity function fit, which confirms that the spin-coated material exhibits anisotropy.

### Coupled oscillator model

The coupled oscillator model presented by solid lines in Fig. 2 (B and C) was fitted to the experimental data independently for measurements in horizontal and vertical polarization.

For the horizontal polarization (Fig. 2B) of the polariton modes, the 4 × 4 Hamiltonian solution is

$${\hat{H}}_{H}=(\begin{array}{cccc} E_{\chi } & \Omega_{H_{1}}/2 & \Omega_{H_{2}}/2 & \Omega_{H_{3}}/2\\ \Omega_{H_{1}}/2 & E_{\phi H_{1}}(k) & 0 & 0\\ \Omega_{H_{2}}/2 & 0 & E_{\phi H_{2}}(k) & 0\\ \Omega_{H_{3}}/2 & 0 & 0 & E_{\phi H_{3}}(k) \end{array})$$ (6)

Fitting with exciton energy *E*_χ_ = 2.350 eV resulted in coupling strengths of Ω_*H*_1__ = 67.2 meV, Ω_*H*_2__ = 88.4 meV, and Ω_*H*_3__ = 94.4 meV.

For the vertical polarization (Fig. 2C) of the polariton modes, the 3 × 3 Hamiltonian solution is

$${\hat{H}}_{V}=(\begin{array}{ccc} E_{\chi } & \Omega_{V_{1}}/2 & \Omega_{V_{2}}/2\\ \Omega_{V_{1}}/2 & E_{\phi V_{1}}(k) & 0\\ \Omega_{V_{2}}/2 & 0 & E_{\phi V_{2}}(k) \end{array})$$ (7)

Fitting with the same exciton energy leads to coupling strengths of Ω_V_1__ = 78.3 meV and Ω_V_2__ = 108.7 meV.

Dispersion relations of uncoupled photonic modes *E*_ϕV/H*_i_*_ are marked in Fig. 2 (B and C) with white dashed lines.

### Berreman method

The spectra presented in Fig. 2 (E and F) show the reflectance calculated for H and V incident light polarization using the Berreman method (*62*). Simulated cavity consists of two DBR mirrors made of five pairs of SiO_2_ and TiO_2_ layers calculated for a maximal reflectance at 530 nm. The space between the mirrors consists of an LC part and a 120-nm-thick perovskite layer directly on top of the SiO_2_ layer of DBR. To match the position of the cavity modes with the experiment, the LC layer is separated into three parts: two interface layers described by an isotropic ordinary refractive index *n*_o_ with a thickness of 170 nm and a central anisotropic LC layer with a thickness of 845 nm described by a diagonal dielectric tensor with the extraordinary refractive index *n*_e_ along the *x* direction and *n*_o_ for the two remaining directions. *n*_o_ and *n*_e_ values of the LC are based on (*47*), and refractive indexes of the perovskite layer are presented in Fig. 2D.

In the simulations shown in Fig. 4 (C, E, K, L, and M), the total length of the LC cavity is equal to *L* = 1362 nm. The twisted nematic orientation of the LC molecules around the *z* axis occurs within distances *l* = 68 nm at the DBR/LC interface with a total of 45° reorientation between the top and bottom DBR. Simulations shown in Fig. 4 were performed with an additional rotation of LC by 42.5° around the *y* axis. Simulations were performed with five SiO_2_/TiO_2_ DBR pairs centered at 555 nm.

The dispersion of the cavity modes obtained from the Berreman method was fitted with Eq. 5 with parameters $E_{\phi }^{0}$ = 2.0997 eV, ∆_HV_ = 13.3 meV, ∆_AD_ = −14.9 meV, *m_x_* = 1.27 × 10^−5^ *m_e_*, *m_y_* = 1.38 × 10^−5^ *m_e_*, δ*_x_* = −0.32 meV μm^2^, δ*_y_* = −0.53 meV μm^2^, δ*_xy_* = 0.35 meV μm^2^, and α = −2.13 × 10^−3^ eV μm.

Calculations presented in Fig. 5 (A to F) were performed for the same parameters but with ∆_AD_ = 18.9 meV observed on the sample with 30° rubbing disorientation (see the Supplementary Materials).

To calculate the Berry curvature, we follow the procedure described in (*41*). On the basis of the Stokes parameters, we define the following angles

$$\Theta (k)=\text{arccos}\;S_{3}(k)$$ (8)

$$\Psi (k)=\text{arctan}\frac{S_{2}(k)}{S_{1}(k)}$$ (9)

and calculate the Berry curvature as

$$B_{z}=\frac{1}{2}\text{sin}\Theta (\partial_{k_{x}}\Theta \partial_{k_{y}}\Psi -\partial_{k_{y}}\Theta \partial_{k_{x}}\Psi )$$ (10)
