# Supplementary material for: Electrically tunable Berry curvature and strong light-matter coupling in liquid crystal microcavities with 2D perovskite
Source: Sci Adv. 2022 Oct 5;8(40):eabq7533. doi: 10.1126/sciadv.abq7533 (PMC9534495; doi:10.1126/sciadv.abq7533)
Supplement: Supplementary file 1 — Supplementary Text Figs. S1 to S10 [file sciadv.abq7533_sm.pdf]

Science Advances
AAAS

Supplementary Materials for

Electrically tunable Berry curvature and strong light-matter coupling in liquid crystal microcavities with 2D perovskite

Karolina Łempicka-Mirek *et al.*

Corresponding author: Barbara Piętka, barbara.pietka@fuw.edu.pl

Sci. Adv. **8**, eabq7533 (2022)
DOI: 10.1126/sciadv.abq7533

The PDF file includes:

Supplementary Text
Figs. S1 to S10
Legend for movie S1

Other Supplementary Material for this manuscript includes the following:

Movie S1

Supplementary Text

Energy tuning with increasing voltage

Figure S1 presents angle-resolved photoluminescence spectra for increasing voltage applied to the cavity with homogeneous LC orientation, corresponding to the data at normal incidence presented in Fig. 3C in the main text.

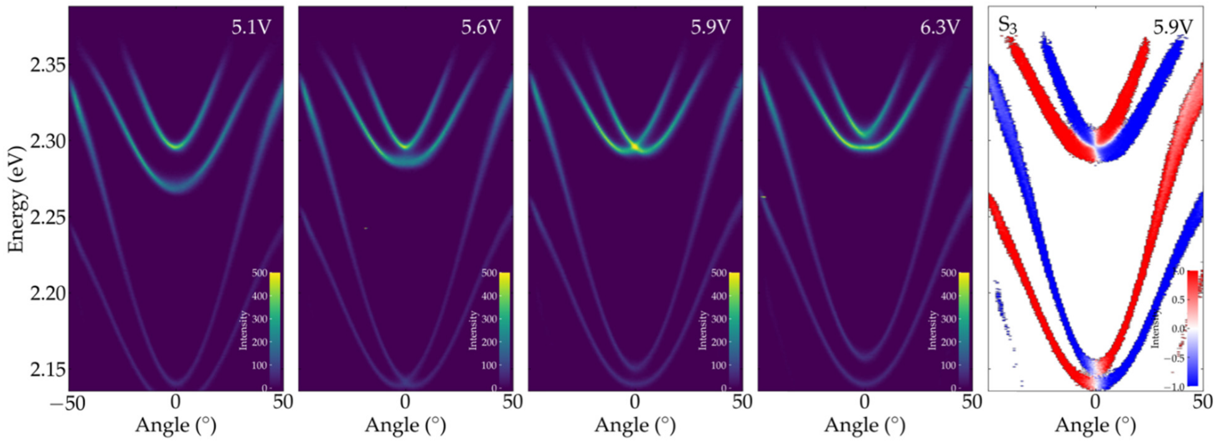

Fig. S1. Energy tuning in liquid crystal filled microcavity with 2D-perovskite.

Parameters for simulations with helical LC structure

In the Berreman matrix model in Fig. 4, the total length of the LC cavity L = 1362 nm. Rotation of LC molecules around the z axis occurs within a distance of l = 68 nm from each of the DBR/LC interfaces, as shown schematically in Fig. S2. The simulations presented in Fig. 4 in the main article were performed for all directors of LC structures rotated by 42.5 deg from the y axis, with 5 SiO_2/TiO_2 DBR pairs centered at 555 nm.

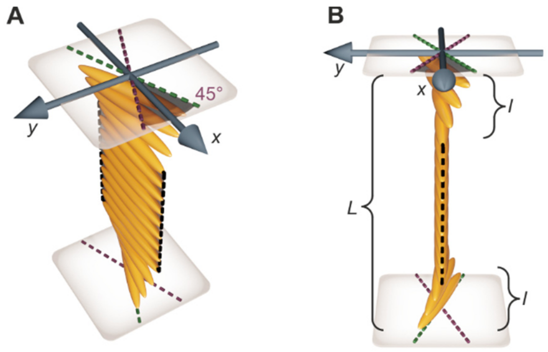

Fig. S2. Scheme of helical LC structure. (**A**) Illustrative graphics of an arrangement of the director (here represented with ellipsoids of revolution) which rotates by 45 deg to each other around z axis. (**B**) Within the full cavity length L, rotation of molecular director occurs within a distance of l from each of the LC/DBR interfaces.

Coupling strength in LCMC

The dispersion of the electric permittivity of the perovskite PEPI based on ellipsometric measurement can be obtained with a good agreement approximated with a simple Lorentz model of excitonic transition given by formula:

$$\varepsilon(E) = \varepsilon'(E) + \varepsilon''(E) = \varepsilon_0 - \frac{f}{E_{ex}^2 - E^2 - \mathrm{i}\,\gamma_{ex}\,E}. \qquad \text{(S1)}$$

Fitting of Eq. S1 to the experimental data is shown in Fig. S3, with fitted parameters $\varepsilon_0 = 4.107$, $f = 0.712\ (\mathrm{eV})^2$, $\gamma_{ex} = 40.4$ meV and $E_{ex} = 2.396$ eV (517.49 nm).

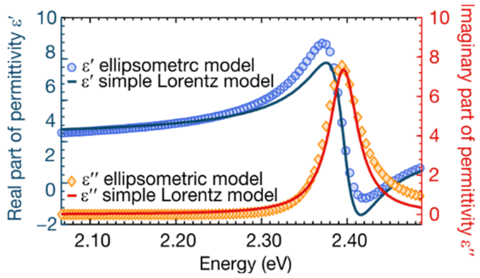

Fig. S3. Real (ε′) and imaginary (ε″) part or dielectric function fitted with Lorentz model.

The obtained approximation of the optical properties of the perovskite layer can be further used to analyze the dependence of exciton-cavity photon coupling in the LC microcavity on the mode polarization and the cavity thickness. Fig. S4 presents angle-resolved reflectance spectra simulated by the Berreman method for a cavity containing a 20 nm perovskite layer of LC various thicknesses. The simulations were performed for the extraordinary direction of LC along the *x* axis, corresponding to the experiment without external voltage.

The calculations were performed for the thicknesses of LC slab resulting in zero energy detuning between the excitonic transition in the perovskie and the photonic mode at normal incidence. Behavior of the uncoupled photonic mode can be determined by taking the dielectric function of the perovskite layer without excitonic transition i.e. $\varepsilon(E) = \varepsilon_0$. Dispersions of such "empty" cavities are marked by dotted white lines in Fig. S4.

For known exciton and bare cavity mode energy and normal incidence, the exciton-photon coupling strength in simple 2×2 coupled oscillator model can be determined based on energy of the polaritonic modes. Results of such an approach are marked in Fig. S4 by color dashed lines. For small thicknesses of LC layers and for low incidence angles, the results of such model with good agreement match the simulated reflectance spectra. Obtained coupling strengths are plotted in Fig. S5. The observed coupling strengths for both polarization modes decrease with the LC layer thickness. Additionally, for a fixed cavity width, the coupling strength is higher for the vertically polarized mode, as the lower ordinary refractive index results in lower mode volume compared to the higher extraordinary refractive index of the horizontally polarized mode.

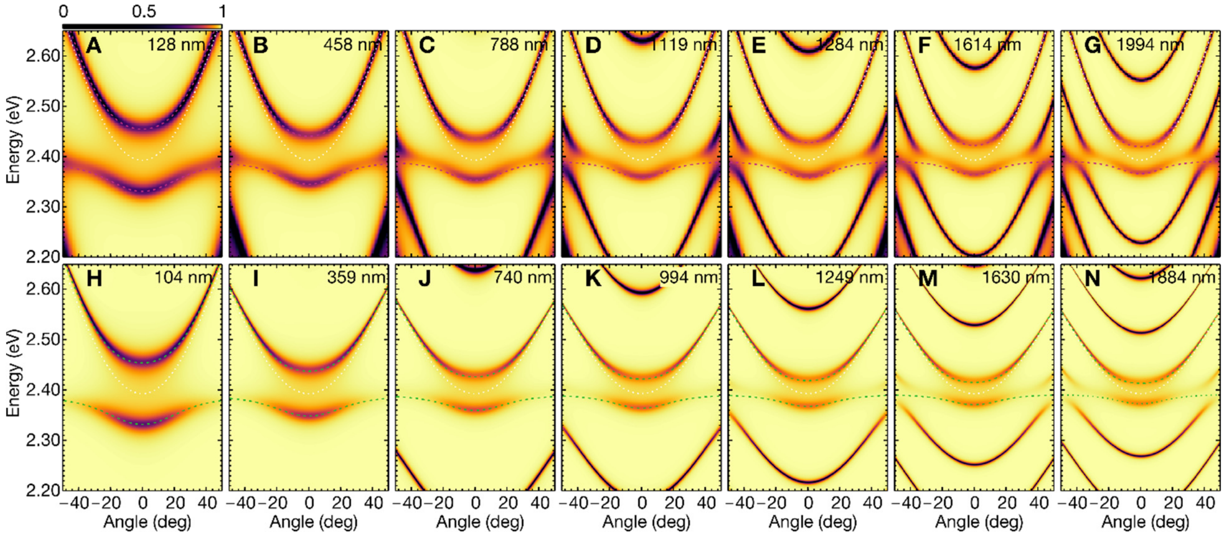

Fig. S4. Simulated angle-resolved reflectance spectra for increasing LC layer thickness. The dashed color lines show the dispersion relation from 2×2 coupled oscillator model, based on the dispersion of the uncoupled cavity marked by white dotted lines. (**A**–**G**) Vertical polarization, (**H**–**N**) horizontal polarization.

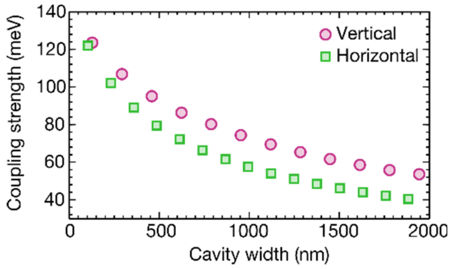

Fig. S5. Exciton-cavity photon coupling strength dependence on LC microcavity thickness for modes polarized horizontally (n_e) and vertically (n_o).

Berry curvature tuning

Polarization resolved transmission measurement was performed for the sample with 30 deg disorientation of the rubbing layers. Angle-resolved photoluminescence maps at different voltages collected on this sample are presented in Fig. S6, whereas voltage tuning at normal incidence in photoluminescence and transmission measurements are shown in Fig. S7. The investigated range of voltages applied to the cavity presented in Fig. 5 corresponded to HV detuning values between −15 meV and 30 meV, as shown in Fig. S8 calculated for Δ_{AD} = 18.9 meV. Berry curvature extracted from the polarization of the lower energy mode at different voltages is shown in Fig. S9 (A to G) and compared with the theoretical calculations in Fig. S9 (H to N).

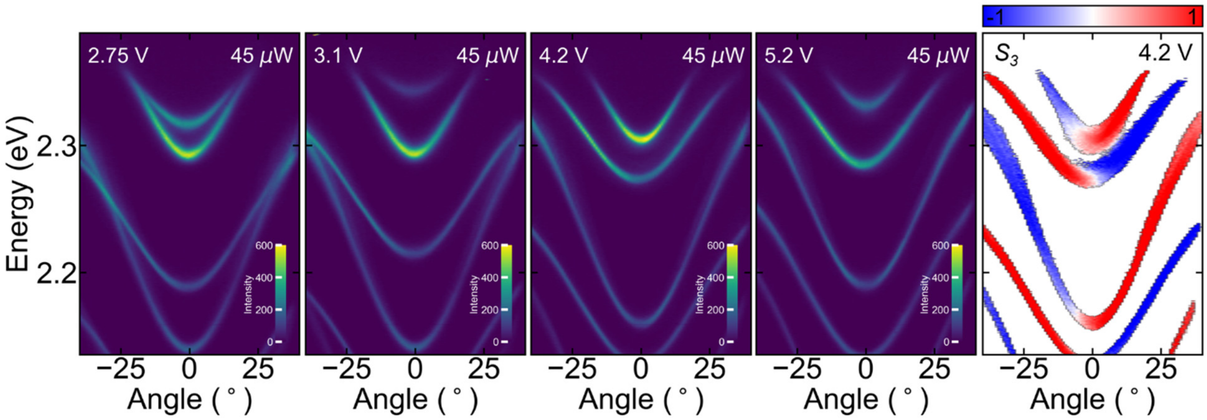

Fig. S6. Energy tuning in liquid crystal filled microcavity with 2D-perovskite with 30 deg oriented LC layer.

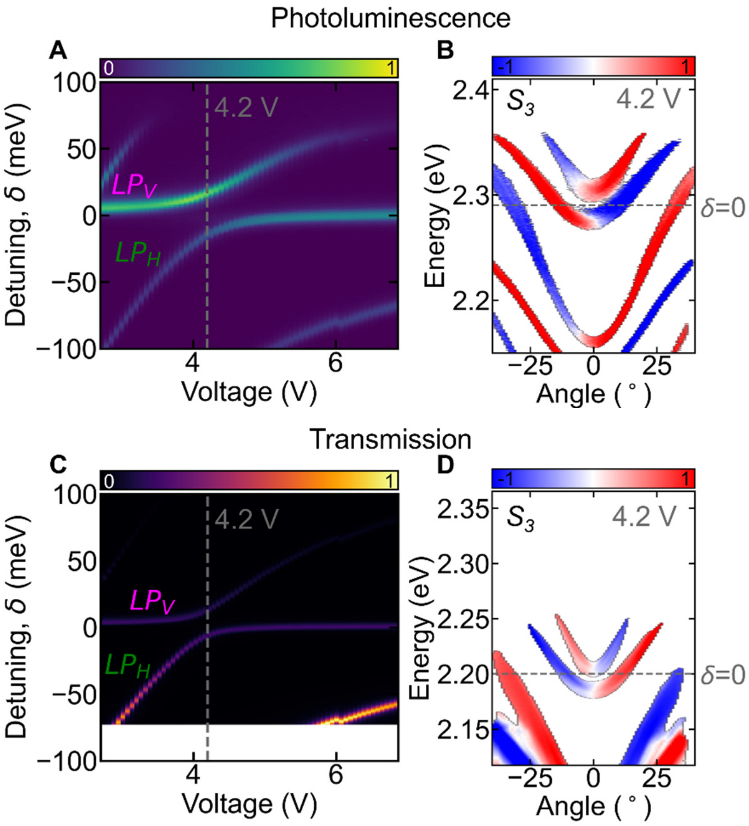

Fig. S7. Gap opening in the Rashba-Dresselhaus (RD) regime for microcavity with 30 deg oriented LC layer. (**A**,**C**) Measured energy resolved (A) photoluminescence and (C) transmission at k = 0 versus applied voltage for diagonal polarization with gap opening. (**B**,**D**) Measured angle-resolved (B) photoluminescence and (D) transmission spectra in R-D regime for S_3 polarization with gap opening.

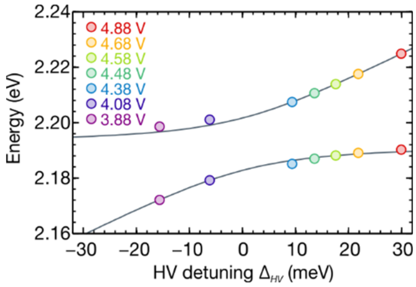

Fig. S8. Calculated energy of the photonic modes at normal incidence for increasing HV detuning (gray lines) compared with experimental results for varying voltage applied to the LCMC (circles).

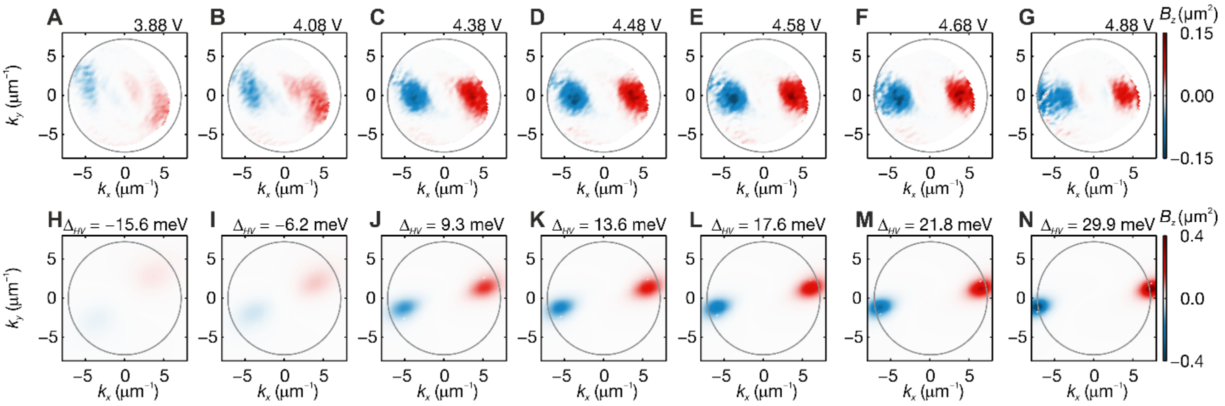

Fig. S9. Electrically tunable photonic Berry curvature. (**A–G**) Berry curvature distribution for lower energy mode with increasing voltage applied to the LCMC extracted from the polarization resolved transmission measurement. Gray circles mark experimental region limited by numerical aperture of the objectives. (**H–N**) calculated Berry curvature for HV detunings corresponding to the experimental results.

Experimental set-up

The scheme of the experimental setup used for the optical measurements is presented in Fig. S10.

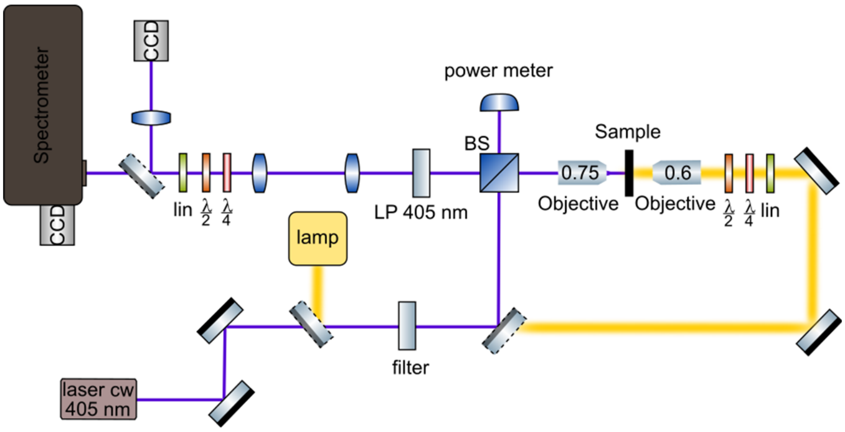

Fig. S10. Scheme of experimental set-up in both configuration: reflectance and transmission.

Movie S1. Tuning of photonic Berry curvature with external voltage and corresponding calculations.
